# Ten-year experience of more than 35,000 orofacial clefts in Africa

**DOI:** 10.1186/s12887-015-0328-5

**Published:** 2015-02-14

**Authors:** Julia C Conway, Peter J Taub, Rochelle Kling, Kurun Oberoi, John Doucette, Ethylin Wang Jabs

**Affiliations:** 1 Department of Pediatrics at Icahn School of Medicine at Mount Sinai, One Gustave L Levy Place, Box 1497, New York, NY 10029 USA; 2 Department of Surgery at Icahn School of Medicine at Mount Sinai, New York, NY USA; 3 Department of Dentistry at Icahn School of Medicine at Mount Sinai, New York, NY USA; 4 Department of Preventive Medicine at Icahn School of Medicine at Mount Sinai, New York, NY USA; 5 Department of Genetics and Genomic Sciences at Icahn School of Medicine at Mount Sinai, New York, NY USA; 6 Developmental and Regenerative Biology at Icahn School of Medicine at Mount Sinai, New York, New York, USA, New York, NY USA; 7 State University of New York Downstate Medical School, Brooklyn, NY USA; 8 Johns Hopkins University Medical School, Baltimore, MD USA

**Keywords:** Cleft, Lip, Palate, Epidemiology, Africa

## Abstract

**Background:**

Surgical correction of orofacial clefts greatly mitigates negative outcomes. However, access to reconstructive surgery is limited in developing countries. The present study reviews epidemiological data from a single charitable organization, Smile Train, with a database of surgical cases from 33 African countries from 2001–2011.

**Methods:**

Demographic and clinical patient data were collected from questionnaires completed by the participating surgeons. These data were recorded in Excel, analyzed using SPSS and compared with previously reported data.

**Results:**

Questionnaires were completed for 36,384 patients by 389 African surgeons. The distribution of clefts was: 34.44% clefts of the lip (CL), 58.87% clefts of the lip and palate (CLP), and 6.69% clefts of the palate only (CP). The male to female ratio was 1.46:1, and the unilateral: bilateral ratio 2.93:1, with left-sided predominance 1.69:1. Associated anomalies were found in 4.18% of patients. The most frequent surgeries included primary lip/nose repairs, unilateral (68.36%) and bilateral (11.84%). There was seasonal variation in the frequency of oral cleft births with the highest in January and lowest by December. The average age at surgery was 9.34 years and increased in countries with lower gross domestic products. The average hospital stay was 4.5 days. The reported complication rate was 1.92%.

**Conclusions:**

With the exception of cleft palates, results follow trends of worldwide epidemiologic reports of 25% CL, 50% CLP, and 25% CP, 2:1 unilateral:bilateral and left:right ratios, and male predominance. Fewer than expected patients, especially females, presented with isolated cleft palates, suggesting that limitations in economic resources and cultural aesthetics of the obvious lip deformity may outweigh functional concerns and access to treatment for females. A fewer than expected associated anomalies suggests either true ethnic variation, or that more severely-affected patients are not presenting for treatment. The epidemiology of orofacial clefting in Africa has been difficult to assess due to the diversity of the continent and the considerable variation among study designs. The large sample size of the data collected provides a basis for further study of the epidemiology of orofacial clefting in Africa.

**Electronic supplementary material:**

The online version of this article (doi:10.1186/s12887-015-0328-5) contains supplementary material, which is available to authorized users.

## Background

Limited access to reconstructive surgery in developing countries has led to the involvement of international organizations that provide surgical correction for patients with orofacial clefts. Based in New York City, the Smile Train organization offers training and financial support for physicians and institutions to provide surgical procedures for patients with clefts of the lip and/or palate. Its goal is to enhance care by local physicians and build infrastructure in developing countries rather than conduct missions. In 15 years of operation, the Smile Train organization enabled repair of over 1,000,000 clefts in 87 countries, and many of these repairs have been in the African continent [1].

Orofacial clefting is associated with elevated infant mortality and significant morbidity in many developing nations where barriers to ensuring multidisciplinary treatment still remain. A cleft of the palate is associated with feeding difficulties in infancy, chronic otitis media due to eustachian tube dysfunction, midface hypoplasia, hypernasality of speech and difficulties with articulation and language development. The possible sequelae of undetected hearing loss can be socially isolating and compound challenges with communication. Another problem of clefts of the lip (CL) is the physical deformity and the associated social and psychiatric morbidity [2,3]. The stigma of an unrepaired orofacial cleft greatly alters a child’s ability to integrate into the social and cultural environment. Beyond the aesthetic deformity, orofacial clefts are given a wide variety of meanings and consequences in different cultures. In regards to etiology, some groups view clefts to be due to divine will, evil spirits, handling sharp objects during an eclipse, or even a husband fishing during the pregnancy [4,5]. In Nigerian society, it is sometimes viewed as divine punishment for parental sins such as witchcraft or prostitution, and the children are therefore kept away from the public [3]. Many of these misconceptions affix blame to parents and families, further isolating the child within their own family and community, and complicating access to complete medical and surgical care.

Clefts also can be associated with a variety of other deformities or syndromes. Approximately 30% of clefts are syndromic [6]. Syndromic cases are associated with defects including musculoskeletal, cardiovascular and central nervous system anomalies [7]. Clefts of both the lip and palate are noted to present with associated malformations: 34% of patients with clefts of the lip and palate (CLP) [6], 14-20% of CL patients [6,7], and as much as 47% of patients with isolated cleft palate (CP) [7]. Non-syndromic orofacial clefting is multifactorial, associated with over 70 candidate genes and chromosomal aberrations, as well as environmental factors [8].

Clefts are associated with teratogens like maternal use of alcohol, smoking [9], and prenatal nutrition, vitamin B6 and folate [10]. Drugs such as retinoic acid and anticonvulsants, infections, ionizing radiation, toxins, occupational exposures, maternal obesity and hyperthermia have also been thought to have an effect [8]. In Zambia and China, seasonal variation showed a peak in cleft births in the summer months [11,12], and herbal medications in Nigeria were similarly associated [13]. Both genetics and environment are believed to play a role in the development of clefts, which result from abnormal embryologic development of the frontonasal and maxillary prominence as well as the palatal shelves to fuse and form the lip, the palate, or both.

Orofacial clefting is the most common congenital malformation of the head or neck [14]. Worldwide, the birth prevalence is 1/700 live births [15]. Prevalence is highest in Asians (1/500), intermediate in Caucasians (1/1,000), and lowest in African populations (1/2,500) [8,15-18]. Clefts of the lip have a 2:1 male to female ratio, while clefts of the palate have a 1:2 male to female ratio [15]. Clefts of the lip are more commonly unilateral than bilateral and favor the left side. For all ethnicities together, approximately 50% of all clefts are combined clefts of the lip and palate, 25% to 35% involve the lip only [19,20], and isolated CP accounting for approximately 25% [14,19,21,22].

While the epidemiology of orofacial clefting in Africa has been studied, results vary widely between publications. African populations are unique not just in lower incidence (1/2,500) [11,15,23,24], but also in cleft type distribution. Individual African studies have reported lower than worldwide percentages of CP (4% [11,25], 6-8% [26], 12% [27], and 13% [28]). This decreased percentage of isolated clefts of the palate has been attributed to the increased stigma associated with clefts of the lip, causing greater concern to patients and families. A cleft of the palate may not be as noticeable or considered as important [29]. Many patients also cannot afford a second surgery so ensuring that patients return after lip repair for palatoplasty has been difficult [30,31].

Greater information regarding these obstacles to care, as well as understanding true epidemiological patterns of orofacial clefting in Africa are crucial to shaping future treatment efforts. The present study analyzes 36,384 orofacial cleft patients over a 10-year period in 33 different African countries. In the context of studies of cleft epidemiology in Africa, this large patient cohort makes a contribution toward standardizing and understanding epidemiologic trends of orofacial clefting on this diverse continent.

## Methods/design

A retrospective review of patients who underwent surgery to repair an orofacial cleft in Africa between 2001 and 2011 was performed. Demographic and clinical data were collected from electronic questionnaires completed by surgeons and healthcare providers using Smile Train Express, an online medical record database. Through partnerships with Smile Train, surgeries performed in Zambia by the organization “Interplast”, now named ReSurge International, and in Kenya by “Gertrude’s Garden” were included as 753 paper records.

The Icahn School of Medicine at Mount Sinai’s IRB approved this study. Deidentified data analyzed included the country, hospital, surgeon, age, gender, family history, maternal factors, pregnancy complications, associated anomalies; cleft anatomic location (lip, alveolus, hard palate, soft palate), and surgical complications. Cleft types were classified according to the International Classification of Diseases (ICD9) diagnosis codes [32], which utilizes three general groups of CL, CLP and CP. These were further categorized by laterality and completeness. These data sets were recorded and entered into Excel (Microsoft, Raymond, WA), and descriptive statistics were performed using the Statistical Package for the Social Sciences version 20.0 (SPSS Inc., Chicago, Ill.).

## Results

### General characteristics

A total of 389 surgeons performed surgery on 36,384 patients from 2001 to 2011 in 33 countries of the African continent. Most procedures were performed in nine East African countries (68.3%) (Table 1). Even though data were collected beginning in 2001 and continued through 2011, 86.1% of the surgeries were performed between 2008 and 2011 (Additional file 1). Racially, the majority of patients (96.8%) self-identified as Black (Additional file 1). Only 0.1% of patients were made aware of Smile Train through the internet. The majority of patients learned of Smile Train through other charity organizations (36.6%), hospitals or physicians (23.3%), or friends and relatives (18%). The average age of patients at surgery was 9.34 years (Table 2). The average age varied among regions with the lowest average age of 3.63 years in the South and the highest average age of 11.22 years in the Central region (Table 1). The average age interestingly did not decrease with time but increased to approximately 9 to 10 years of age for the last five years of study (Additional file 1).

**Table 1:** **Chronologic and geographic distribution of cleft cases in Africa**

Region of Africa	Timeframe of data collection	Percent of cases*	Participating countries	Average age of cases at surgery (years)
Central	2004-2011	2.0	Cameroon, Democratic Republic of the Congo	11.22
North	2006-2011	2.2	Egypt, Sudan, South Sudan	5.26
South	2001-2011	4.6	Malawi, Mozambique, South Africa, Zambia, Zimbabwe	3.63
West	2002-2011	22.2	Benin, Burkina Faso, Côte D’Ivoire, Gambia, Ghana, Guinea, Liberia, Mali, Mauritania, Niger, Nigeria, Senegal, Sierra Leone, Togo	8.50
East	2002-2011	68.3	Burundi, Djibouti, Ethiopia, Kenya, Madagascar, Rwanda, Somalia, Tanzania, Uganda	10.66

*The total number of patients with a recorded geographical region is 36,357.

**Table 2:** **Characteristics of the patients undergoing each type of surgery**

Type of surgery	Number*	Percent	Average age at surgery (years)	M:F ratio**	Hospital stay length (days)
Primary lip/ nose U/L**	19,058	68.36	9.90	1.48	4.27
Primary lip/ nose B/L**	3,302	11.84	8.83	1.63	5.04
Lip/ nose revision	1,168	4.19	14.04	1.03	4.53
Primary cleft palate	3,193	11.45	6.39	1.21	5.29
Secondary cleft palate	282	1.01	9.05	0.99	3.31
Fistula repair	545	1.95	8.35	1.15	4.50
Alveolar bone graft	42	0.15	13.2	0.68	4.96
Other	290	1.04	8.23	1.13	4.6
Total*	27,880	100	9.34	1.42	4.5

*The total number of patients with a recorded type of surgery is 27,880.

**Abbreviations: *M:F ratio* male: female ratio, *U/L* unilateral, *B/L* bilateral.

Of maternal factors recorded, the most common factor was a complication during pregnancy, which occurred in 2.3% of mothers (Table 3). It is reasonable to expect that orofacial clefts may show seasonal variations in date of birth based on factors such as maternal malnutrition and low intake of folic acid [33]. The present data showed the greatest number of patients born in January (12.97%) and the fewest born in December (5.25%) (Table 4) with a decline in numbers over the course of the year. The remainder of the months showed little variation, with fall (Sept, Oct, Nov), having the fewest number of cleft births (21.12% vs. 26.32% winter, 25.82% spring, 25.72% summer).

**Table 3:** **Pregnancy and environmental factors**

Maternal factor	Positive history*	Percent	Negative history*	Percent	Uncertain history*	Percent
Mother Smoked	284	0.8	34,901	95.9	1,198	3.3
Birth Complications	576	1.6	34,279	94.2	1,528	4.2
Mother Consumed Alcohol	797	2.2	34,244	94.1	1,342	3.7
Pregnancy Complications	823	2.3	34,037	93.5	1,523	4.2

*The total number of patients with recorded information is 36,383.

**Table 4:** **Distribution of the number of cleft patients born each month**

Month	All African patients		Zambian patients	
	Total	%*	Total	%*
January	4720	12.97	96	9.28
February	2944	8.09	100	9.66
March	3370	9.26	88	8.50
April	3285	9.03	110	10.63
May	3104	8.53	98	9.47
June	3362	9.24	102	9.86
July	3227	8.87	68	6.57
August	2772	7.62	77	7.44
September	2621	7.20	78	7.54
October	2686	7.38	81	7.83
November	2380	6.54	76	7.34
December	1913	5.25	61	5.89

*36,384 patients had a birth month recorded, and 1,035 patients born in Zambia had a birth month recorded.

### Classification of clefts and gender

The classification system used was based on the ICD9 diagnostic codes, reclassified from the data provided by Smile Train. With the reformatted classification, 35,323 of the 36,384 patients had a recorded cleft type. A small percentage (2.92%) of patient data contained missing or erroneous information and could not be transferred to the clinical codes due to ambiguity. These were not considered in the cleft distribution. Alveolar defects were considered with palatal clefts. The anatomic cleft type distribution was 34.44% CL, 58.87% CLP, and 6.69% CP, or 5.1:8.8:1.0 ratio. The total unilateral:bilateral ratio was 2.93:1 with a skewed unilateral:bilateral ratio of CL of 18.16:1. Total left-sided clefts predominated over the right-sided cleft with a ratio of 1.69:1 (Table 5). Male patients were more frequent with an overall male to female ratio of 1.46. For CL and CLP, the male to female ratios were higher at 1.65 and 1.44, respectively. Females predominated for CP with a male to female ratio of 0.88, but less than typically observed in developing countries (Table 5).

**Table 5:** **Analysis of the distribution of cleft type**

Type of cleft*	Number**	Percent	M:F ratio	U/L:B/L ratio	L:R ratio
**Total CL**┼	**12,166**	**34.44**	**1.65**	**18.16**	**1.70**
UCCL, left	1,711	4.84	1.32		
UCCL, right	1,082	3.06	1.13		
UICL, left	5,555	15.73	1.81		
UICL, right	3,183	9.01	1.84		
BCCL	523	1.48	1.49		
BICL	112	0.32	1.87		
**Total CLP**	**20,795**	**58.87**	**1.44**	**2.12**	**1.67**
UCCLP, left	2,328	6.60	1.42		
UCCLP, right	1,407	3.97	1.46		
UICLP, left	6,517	18.45	1.28		
UICLP, right	3,884	11.00	1.32		
BCCLP	1,932	5.47	1.71		
BICLP	4,727	13.38	1.70		
**Total CP**	**2,362**	**6.69**	**0.88**	**0.39**	**1.58**
UCCP, left	38	0.11	1.24		
UCCP, right	13	0.03	2.25		
UICP, left	366	1.04	1.23		
UICP, right	243	0.69	1.59		
BCCP	25	0.07	1.78		
BICP	1,677	4.75	0.73		
**Total**	**35,323**	**100**	**1.46**	**2.93**	**1.69**

*Cleft type, which is classified according to ICD9 diagnostic codes [32], as well as sex ratios, unilateral to bilateral and left to right ratios were calculated using a denominator of the 35,323 patients with complete, valid entries recorded.

**The total number of patients with a recorded cleft type is 35,323.

┼*Abbreviations*: *M:F* male to female ratio, *U/L* unilateral, *B/L* bilateral, *L:R* left to right ratio, *UCCL* unilateral complete cleft of the lip, *UICL* unilateral incomplete cleft of the lip, *BCCL* bilateral complete cleft of the lip, *BICL* bilateral incomplete cleft of the lip, *UCCLP* unilateral complete cleft of the lip and palate, *UICLP* unilateral incomplete cleft of the lip and palate, *BCCLP* bilateral complete cleft of the lip and palate, *BICLP* bilateral incomplete cleft of the lip and palate, *UCCP* unilateral complete cleft of the palate, *UICP* unilateral incomplete cleft of the palate, *BCCP* bilateral complete cleft of the palate, *BICP* bilateral incomplete cleft of the palate.

### Associated anomalies and family history

Of the total patients with a recorded cleft type, 4.18% reported an associated anomaly. The distribution of patients with one or more associated anomalies by anatomic location was 5.52% for CLP patients, 5.38% for CP patients, and 1.65% for CL patients. Of the associated anomalies, growth abnormalities were the most common (30.8%) (Table 6). Among patients with associated anomalies, there was an overall male predominance (male to female ratio 1.38:1). The sole exception was for mandibular anomalies, which demonstrated a slight female predominance (male to female ratio 0.89:1). Patients with clefts of the lip and palate also had greater percentages of affected immediate and distant relatives, 3.54% and 3.82%, respectively (Table 6). Of the 5,198 patients who reported having prior surgery (14.72%), 3,797 patients (73.05%) reported prior repair of a cleft lip, 954 (18.35%) reported prior repair of a lip and palate, and 308 (5.93%) reported prior palate repair alone.

**Table 6:** **Characteristics of patients with associated anomalies, and the percentage of patients with affected relatives**

Associated anomaly	Number	Percent	M:F ratio*
Growth	666	30.8	1.31
Eyes	354	16.4	1.28
Fingers/Toes	176	8.2	1.32
Skull	159	7.4	1.24
Limbs	147	6.8	1.45
Ears	142	6.6	1.29
Mental	125	5.8	1.16
Mandible	115	5.3	0.89
Heart	84	3.9	1.10
Skin	80	3.7	1.35
Urinary	61	2.8	2.05
Tongue	50	2.3	1.38
**Total**	2,159**	**4.18 of total**	**1.38**
		1.65% of CL	
		5.38% of CP	
		5.52% of CLP	
**Cleft type**	**Percent with immediate relative affected**		**Percent with distant relative affected**
CL	1.79		2.42
CLP	3.54		3.82
CP	3.47		2.88
**Total**	**2.93**		**3.27**

*Abbreviation: *M:F ratio* male: female ratio.

**2,159 is the total number of anomalies seen, not the number of patients. Multiple anomalies found in one patient are considered individually. 6,546 is the number of anomalies when nose and speech defects are considered associated anomalies. In this review, they are considered related to the original defect.

### Surgical procedures

The number of patients with recorded surgical type was 27,880. The majority (68.36%) underwent primary repair of a unilateral cleft lip/nose deformity. Each of the other procedures occurred in less than 12% of reported cases and included primary repair of a bilateral lip deformity, lip/nose revision, primary repair of a cleft palate, secondary cleft palate surgery, fistula repair, alveolar bone grafting, and other. The average length of admission was 4.5 days (Table 2). The primary technique of unilateral lip repair was rotation advancement that was performed on 17,608 of 20,040 patients who underwent unilateral lip surgery (87.86%). The primary method of bilateral lip repair employed straight line repair (2,027 of the 3,574 patients, 56.72%). The primary method of palate repair was the Langenbeck (1,972 of 3,946 patients, 49.98%) (Table 7).

**Table 7:** **Number, gender ratio, and complication rate of each repair type**

Type of repair	Number	Percent	M:F ratio**	Percent complications
**Unilateral lip repair techniques**				
Rotation advancement	17,608	87.86	1.48	0.91
Triangular	1,318	6.58	1.58	0.99
Other	1,114	5.56	1.34	1.7
Sub-total	20,040	72.71		
**Bilateral lip repair techniques**				
Straight line	2,027	56.72	1.51	2.2
Forked flap	794	22.22	1.31	3.7
Other	753	21.07	1.16	2.5
Sub-total	3,574	12.97		
**Palate repair techniques**				
Langenbeck	1,972	49.98	1.34	6.3
Pushback	512	12.98	1.26	5.3
Other	1,462	37.05	1.30	3.8
Sub-total	3,946	14.32		
Total*	**27,560**	**100**	**1.44**	**100**

*The total number of patients with a recorded type of repair technique is 27,560.

**Abbreviation: *M:F ratio* male: female ratio.

### Postoperative events

Surgical complications were reported in a total of 536 patients (1.92% of the 27,880 patients with a type of surgery recorded). The complication rate for different repair techniques ranged from 0.91 to 6.3% with higher complication rates for repairs of the palate than the lip. Of these complications, 271 (50.56%) occurred during primary lip repair surgeries. The highest complication rate occurred with the Langenbeck palatal repair technique and the least with rotation advancement for unilateral lip repair (Table 7). The most common complication was an undisclosed type of injury (30.22%). For all complications, 3.17% (17 of the total of 536 complications) involved the return to the operating room for care, 6.90% a breathing problem, 10.63% a fistula, 22.76% a wound dehiscence, and 26.31% a non-categorized complication. Of the 57 fistula complications, 45 (78.95%) occurred during primary and secondary cleft palate surgeries. Of the 122 cases of dehiscence, 75 (61.48%) were during primary lip repair, and 37 (30.33%) occurred during cleft palate repair (Additional file 2).

## Discussion

The present review analyzes data from 36,384 patients with orofacial clefts who received care in 33 African countries through Smile Train. Charity organizations present the potential to study large patient cohorts, but to date, few studies have analyzed this patient base [34]. Prior attempts to acquire large amounts of data regarding orofacial clefting in Africa have been limited. Stigma surrounding facial clefting, a predominance of unrecorded home births, challenges to record-keeping with underreporting especially of patients with cleft palates [29], poor healthcare infrastructure, increased mortality of children with clefts [35,36], and shortages of research funding and resources [37,38] have affected the ability to perform epidemiological research across the continent [39].

More than half of the patients in this study presented after four years of age, with an average age at surgery of 9.34 years (Table 2). This is consistent with previous African studies, and it is suspected that the delay in presentation is a result of lack of access to media and education of treatment options for both parents and the home birth attendants [30]. Our study found that the majority of patients (59.90%) were referred to treatment by other charity organizations or hospitals, further supporting that families may not be aware of how to obtain affordable treatment before contact with outside professionals.

Our data show regional differences in age of surgery varied with the level of economic development. Of the three northern countries, Egypt had a low average age of 3.75 years compared to that of Sudan (16.89 years) and South Sudan (19.85 years). Egypt has had the higher gross domestic product (GDP) of all three countries throughout 2001–2011 [40], and had the majority of northern cases recorded in the database, while Sudan and South Sudan are less developed and still recovering from Africa’s longest-running civil war [41]. This correlation with economic development is also seen in the southern region. South Africa has a GDP that has been consistently within the top three in Africa from 2001–2011, and the average age for surgery was 2.22 years, while other southern countries of Malawi (4.62 years), Mozambique (10.21 years), Zambia (2.62 years) and Zimbabwe (4.84 years) had higher average ages and much lower GDPs [40]. Countries with average ages above 10 years were South Sudan (19.85 years), Sudan (16.89 years), Guinea (13.78 years), Burundi (13.91 years), Ethiopia (13.66 years), Rwanda (13.62 years), Djbouti (11.82 years), Democratic Republic of the Congo (11.24 years), and Mozambique (10.21 years). All of these countries, from 2001–2011, had some of the lowest GDPs in the world [40].

The African cleft distribution was analyzed and a CL:CLP:CP ratio of 5.1:8.8:1.0 was found. European reports have generally shown a distribution of 1:2:1 [14,19,21,22]. The observed decrease in African palatal cases, particularly in females, presenting for surgery could reflect genetic variation in the African population, but more likely is due to increased morbidity and mortality from poor feeding leading to neonatal malnutrition and vulnerability to infectious diseases [35,36]. General societal neglect for those with cleft palate can lead to decreased access to palatal surgery, especially in females [25-31]. Also, there may be less concern for the functional rather than aesthetic deformities of cleft lip. This is evidenced by the difficulty many surgeons face as they attempt to ensure patients return after lip repair for a palatal repair [30]. Some surgeons have considered a single-staged cheiloplasty and palatoplasty in order to ensure repair of as many palatal defects as possible [42]. Proportionally fewer African females than males receiving surgical care may reflect another cultural difference observed in this study from that found in developed countries. However, results from other African reports have been mixed in the predominance of males or females presenting with oral clefts [13,43] or other congenital anomalies [44-46].

The occurrence of associated anomalies was found to be lower than expected in the Smile Train data. Previous European reports have noted approximately 30% of newborns with orofacial clefts to show additional congenital anomalies, 30% of which are thought to occur as multiple anomalies of unknown origin, or as a recognized syndrome [6]. The present study reported only 4.18% of patients with an associated anomaly (Table 6). It is possible that patients with more severe defects have a higher mortality rate, or are not brought to treatment through Smile Train. Cleft repair also could be viewed as ineffective in the face of multiple defects. One large study from Kenya found that 8.2% of patients had associated anomalies in the retrospective branch of their study, but showed 25% in the prospective branch [26], suggesting that the patients are born but not presenting for treatment. It is also important to consider the classification of defects. Deformities of the nose and problems with speech were excluded in this study, and future studies should be standardized as to which anomalies are considered and truly independent.

Generally in the literature, the majority of associated anomalies occur in cases involving clefts of the palate rather than of the lip. In a study of 5,449 cases from 23 European birth registries, anomalies were found in 20.8% of CL cases, but 34.0% of CLP cases [6], and others have found associated anomalies in only 8% of CL, but 22% of CP and 28% of CLP cases [47]. However, in Africa lower numbers have been reported with associated anomalies in 4% of CL patients, 5% of CLP patients, and 6% of CP patients [39]. This trend is seen in the present study, where associated anomalies occurred most frequently in CLP (5.52%) and CP patients (5.38%), and in only 1.65% of CL patients (Table 6). This lower frequency of anomalies for orofacial clefting in Africa again may be due to the lack of screening, underreporting, and access to care for these severe cases.

In this study, the majority of mothers did not report smoking, consuming alcohol, pregnancy complications, or delivery complications (Table 3), suggesting cultural differences or incomplete patient reporting. Seasonal variation for African births with clefts was found that might reflect other maternal complicating factors including nutritional variation and infectious exposures. Seasonal variation in the birth of patients born with clefts of the lip and/or palate was also seen both by a study in Zambia [11] as well by Smile Train’s data from China [12]. In Zambia, births of cleft patients were shown to peak in April and May, with more births from March through August (57.2%) (Table 4) [11]. In China, the peaks were in August and October, with the least number of patients born from November to January [12]. When data from our current study was extracted for patients born only in Zambia (Table 4) and directly compared with the findings of Elliott and colleagues [11], April was also found to have the highest number of cleft births (10.63%), but the overall trend reflected the larger African continental data, with a greater number of patients born at the start of the year, and declining to a low in December (5.89%). Like the continental data, the fall months had the least number of cleft births.

It is thought that despite the important role the environment and teratogens may play, they alone account for only some cases of non-syndromic clefts and multiple genetic factors are also involved in causation [48]. One report based on over 2 million children in Norway found parent–child and sibling-sibling recurrence risks to be the same, and also suggested greater familial predisposition, especially for clefts of the palate, than environmental factors alone [49]. Familial risks are generally thought to be higher with the occurrence of isolated CLP and CP, than for isolated CL [49,50]. For non-syndromic CL ± P (cleft lip with or without cleft palate), the recurrence risk for a subsequent sibling is considered to be 3-5% [49,51]. Similarly in the patients studied here, the recurrence risk for CL patients was found to be 1.79% for an immediate relative and 2.42% for a distant relative, and 3.54% and 3.82% respectively for CLP patients and 3.47% and 2.88% for CP respectively (Table 6).

In total, 536 surgical complications were experienced by 375 patients (1.92%) (Additional file 2). Compared to other studies, the complication rates seen here are low (14.9% in a Nigerian study [30], 10% in a Kenyan study [26], both hospital-based). This could be attributed to reduced follow-up in developing countries [42,52]. Other than anesthesia problems, immediate bleeding, need for transfusions, and immediate dehiscence of incision lines, complications may not be reported. The low numbers might also reflect bias on the part of the reporter/surgeon. A higher complication rate might result in a lower referral rate for cases and thus less income. The financial support provided by Smile Train for different types of cleft surgeries is the same, thus participating doctors may select on which cases to perform surgery depending on the local limitations.

With the low rate of reported surgical and medical complications including infections, it is unclear why there was also a long mean hospital stay of 4.5 days compared with many developed countries, especially with lip repairs predominating [53,54]. Anecdotally the medical teams have reported, in some cases of severe malnutrition or dehydration, pre-operative hospital care is administered prior to surgery thus lengthening the time of stay. In at least some cases, many patients and families will travel to the hospital together from a rural/distant area as a cohort for treatment. In these cases, often all patients will remain at the hospital until the entire cohort has received the surgery and can all travel back home together from a rural/distant area. Additionally, given the difficulty of follow-up care for some families, there are some hospitals where some patients stay longer to monitor for complications if they are concerned the family/patient may not return for a follow-up visit (personal communication). However, questionnaires used by Smile Train did not specifically address these issues.

Here, we presented the characteristics and treatment of the largest reported cohort of orofacial clefting patients in Africa. The patient data analyzed were standardized from information entered by the operating surgeon into a specific questionnaire. However, we recognize the limitations of the extensive data despite careful analysis. The true incidence or prevalence of orofacial clefts is not known from this study. The questionnaire does not capture information prospectively and is biased in that only patients that come for care and receive care are reported. Many patients with severe clefts could not have operations because of lack of safe facilities and staffing as designated by Smile Train’s safety and quality assessments and of patients’ health conditions. A small percentage, 2.92% of patients, were not included in our analysis due to invalid data entries or because some patients with prior repairs were not recorded as having clefts. We noted that some patients who had multiple surgeries may have been entered into the original data multiple times. When the response was “other”, the questionnaire did not give the option for further description. We also reformatted cleft types from anatomical categories to fit the ICD9 diagnosis codes [32] using the SPSS software to have more clinical relevance.

## Conclusion

The current cohort of 36,384 patients, across 33 different countries, in mostly rural and economically disadvantaged areas, does provide a substantial contribution to our current understanding of cleft care in Africa. Important findings include smaller than expected percentages and bias for certain types of patients. There were fewer patients with isolated clefts of the palate, associated anomalies, and complications than expected. It is possible that rather than genetic and environmental variation, patients with isolated cleft palates have a higher mortality rate, are being underreported, have diminished access to care, or have greater cultural concerns about the visible versus functional defects. Patients with associated anomalies may also be underrepresented, which could be due to incomplete screening or that patients too severely affected are not brought for treatment. The fact that the age at time of surgery has not yet decreased may indicate increasing awareness is bringing forward cleft patients of all ages, including the backlog of previously untreated cases, for surgical care. These results highlight vulnerable patient populations for consideration in the management of future large-scale treatment efforts.

## Additional files

Additional file 1:
**Table of number of African patients treated through Smile Train per year and their ethnicities.**

Additional file 2:
**Table of complication type and rate for a surgical type performed by Smile Train partners.**

## Abbreviations

CL: Cleft lip
CP: Cleft palate
CLP: Cleft lip and palate
