# Supplementary material for: Ten-year experience of more than 35,000 orofacial clefts in Africa
Source: BMC Pediatr. 2015 Feb 14;15:8. doi: 10.1186/s12887-015-0328-5 (PMC4342189; doi:10.1186/s12887-015-0328-5)
Supplement: Additional file 1: — Table of number of African patients treated through Smile Train per year and their ethnicities. [file 12887_2015_328_MOESM1_ESM.pdf]

Number of patients treated per year and patient ethnicities

Year	Number*	Percent	Average age (years)	Race	Number**	Percent
2001	3	0.1	0.28	Black	35,218	96.8
2002	101	0.3	2.52	Other	572	1.6
2003	73	0.2	2.15	Asian	311	0.8
2004	163	0.5	3.30	Mixed	206	0.6
2005	416	1.1	4.68	Indian	37	0.1
2006	996	2.7	5.90	White	36	0.1
2007	3,275	9.0	8.80	Latino	4	0.01
2008	5,461	15.0	10.52			
2009	6,671	18.3	9.77			
2010	10,015	27.5	10.61			
2011	9,183	25.3	9.47			

*The total number of patients with a recorded year is 36,357.
**The total number of patients with a recorded race is 36,384.
