# Supplementary material for: Ten-year experience of more than 35,000 orofacial clefts in Africa
Source: BMC Pediatr. 2015 Feb 14;15:8. doi: 10.1186/s12887-015-0328-5 (PMC4342189; doi:10.1186/s12887-015-0328-5)
Supplement: Additional file 2: — Table of complication type and rate for a surgical type performed by Smile Train partners. [file 12887_2015_328_MOESM2_ESM.pdf]

Complication type and rate for a surgical type

Complications	Primary lip repair	Primary cleft palate	Secondary cleft palate	Fistula repair	Lip/nose revision	Alveolar bone graft	Other	Total	Percent of complication type
Return to OR	10	4	1	1	1	0	0	17	3.17
Breathing Problem	29	7	0	0	0	0	1	37	6.90
Fistula	6	41	4	4	2	0	0	57	10.63
Dehiscence	75	28	9	4	2	0	4	122	22.76
Other	73	43	11	5	4	0	5	141	26.31
Injury	78	53	5	3	7	1	15	162	30.22
**Total****	**271**	**176**	**30**	**17**	**16**	**1**	**25**	**536**	**100%**
Percent of complications by surgical type	50.56	32.84	5.60	3.17	2.99	0.19	4.66	100%	

*****The total number of patients with a recorded type of surgery is 27,880 patients.
******The percent of patients with a recorded type of surgery that had a complication was 1.92% (536/27,880).
